# HE4 Serum Levels in Patients with BRCA1 Gene Mutation Undergoing Prophylactic Surgery as well as in Other Benign and Malignant Gynecological Diseases

**DOI:** 10.1155/2017/9792756

**Published:** 2017-01-15

**Authors:** Anita Chudecka-Głaz, Aneta Cymbaluk-Płoska, Aleksandra Strojna, Janusz Menkiszak

**Affiliations:** Department of Gynecological Surgery and Gynecological Oncology of Adults and Adolescents, Pomeranian Medical University, Szczecin, Poland

## Abstract

*Objective*. We assess the behavior of serum concentrations of HE4 marker in female carriers of BRCA1 and assess the diagnostic usefulness of HE4 in ovarian and endometrial cancer.* Methods*. A total of 619 women with BRCA1 gene mutation, ovarian, endometrial, metastatic, other gynecological cancers, or benign gynecological diseases were included. Intergroup comparative analyses were carried out, the BRCA1 gene carriers subgroup was subjected to detailed analysis, and ROC curves were determined for the assessment of diagnostic usefulness of HE4 in ovarian and endometrial cancer.* Results*. Statistically lower serum HE4 and CA 125 levels were observed in BRCA1 gene mutation premenopausal carriers. Occult ovarian/fallopian tube cancer was found 3.6%. Each of those patients was characterized by slightly elevated levels of either CA 125 (63.9 and 39.4 U/mL) or HE4 (79 pmol/L). The ROC-AUC curves were 0.892 and 0.894 for diagnostic usefulness of ovarian cancer and 0.865 for differentiation of endometrial cancer from endometrial polyps.* Conclusions*. Patients with BRCA1 gene mutations have relatively low serum HE4 levels. Even the slightest elevation in HE4 or CA 125 levels in female BRCA1 carriers undergoing prophylactic surgery should significantly increase oncological alertness. The HE4 marker is valuable in ovarian and uterine cancer diagnosis.

## 1. Introduction

In recent years hundreds of proteins have been tested for their importance as markers in cancer diseases. A large part of these studies consisted of experiments involving new markers of ovarian cancer. Despite the use of novel imaging techniques as well as increasingly advanced therapeutic methods, ovarian cancer continues to pose the greatest challenge in gynecological oncology due to the late diagnosis and poor prognosis. As recently as several years ago, the only marker used in clinical practice in ovarian cancer patients was CA125. Only after the studies conducted by Hellström et al. [1] in 2003 and subsequently followed by other authors [2–8], the era of research on a very promising glycoprotein of the four-disulfide core family has begun. The four-disulfide core family is a heterogeneous group of small, acidic, and thermally stable proteins of varied functionalities. Starting from the early 1990s to date, a total of about 200 articles have been published on the use of human epididymis protein 4 (HE4) in ovarian cancer while nearly 4000 articles on the use of CA125 marker have been published to date since the early 1980s. It is therefore clear that further research on HE4 is required before its usefulness raises no scientific doubts. As of today, it appears that HE4 is a sensitive and, first of all, specific marker of malignant epithelial ovarian cancers [9, 10]. Combined with CA125, HE4 offers a useful diagnostic method as a part of ROMA algorithm for prediction of the malignant nature of an ovarian tumor [8, 11, 12]. The marker may be successfully used in the monitoring of ovarian cancer [13]; studies of recent years also suggest a high prognostic potential of HE4 [14]. No screening tests have been developed for ovarian cancer to date. The results of all research trials were negative [15]. The only isolated population subgroup that might benefit from ovarian cancer screening tests is patients with mutations within the BRCA1 and BRCA2 genes and burdened by a family history of ovarian cancer [16, 17]. The behavior of the HE4 marker as well as its usefulness in this group of patients has not been unambiguously determined.

## 2. Materials and Methods

A total of 619 patients, 298 premenopausal and 321 postmenopausal, were included in the study in the period from 2010 to 2014. The age of patients ranged from 18 to 92. The initial study population consisted of BRCA1 gene mutation carriers presenting at the Department of Gynecological Surgery and Gynecological Oncology of Adults and Adolescents for prophylactic bilateral salpingooophorectomy and female patients with the most common pathologies of the genital organ (gynecological tumors, noncancerous ovarian cysts, uterine myomas, adnexitis, and metastatic ovarian tumors). Patients with a history of renal and lung diseases were not included in the study. The study was approved by Ethics Committee of Pomeranian Medical University and all patients signed the informed consent for participation. After the consent, blood was drawn from the patients and subsequently delivered to the central laboratory, where separation of serum and determination of HE4 and CA125 serum levels were performed. All assessments were made immediately, without the need for freezing of the material.

Table 1 presents the characteristics of the study population. The final distribution of patients into individual groups was performed after histopathology test results were available and after a number of patients were excluded from the study due to elevated serum creatinine levels. Besides the primary distribution of patients into 9 study groups presented in Table 1, the following subgroups were identified:

- in group C:
  - patients with endometrial ovarian cysts
  - patients with teratoma tumors
  - patients with hemorrhagic cysts
  - patients with paraovarian lesions
- in group E:
  - serous tumors
  - mucinous tumors
  - cystadenofibroma
- in group G:
  - ovarian gonadal tumors
  - vulvar cancers
  - cervical cancers
- in group R:
  - serous cancers
  - mucinous cancers
  - endometrial cancers
  - clear-cell cancers

Mean HE4 values were compared in individual groups and subgroups, and relationships between HE4 and CA125 levels were analyzed in the study population. Diagnostic usefulness of HE4 was also compared to that of CA125 in ovarian cancer patients relative to female patients with benign ovarian lesions; in addition, diagnostic usefulness of HE4 was compared to that of CA125 in endometrial cancer relative to patients with benign endometrial lesions. All the analyses were carried out in three variants: regardless of hormonal status, in postmenopausal patients and in premenopausal patients. Patients were classified as postmenopausal when the last menstruation occurred more than 12 months ago or if FSH serum levels exceeded 30 U/L.

Incidence of occult ovarian cancers (patients with latent ovarian/fallopian tube cancers were excluded from comparative analysis) and the incidence of breast cancer were analyzed and follow-up examinations were carried out in the group of BRCA1 mutation carriers so as to record new cases of breast cancer or peritoneal cancer. The minimum follow-up period was 1 year.

### 2.1. Marker Analysis

Assays were performed at the Central Laboratory of the Independent Public Hospital.

CA125 was determined with the Architect i2000 assay from Abbott Diagnostics. The normal range was 1–35 U/mL. Serum HE4 concentrations were measured with the Elecsys ECLIA assay from Roche running on the cobas e 601 analyzer. The measurement range was 15.0–1500 pmol/L. Samples exceeding the upper range were diluted with Elecsys Diluent Multiassay. Manufacturer's instructions were followed and control samples were within the normal range. The normal upper limit range for serum was below 70 pmol/L.

### 2.2. Statistical Analysis

The statistical analysis was performed using STATISTICA 9.1 PL program.

The descriptive characteristic of the examined population of patients was prepared, determining minimum, maximum mean, and median values. Also the scatter diagrams of empirical values of markers were plotted, subdivided into the studied groups. The mean/median values in particular groups and subgroups were compared using the nonparametric* U*-test of Mann–Whitney.

In order to determine the relation between the analyzed markers, Pearson's linear correlation coefficients were counted and the linear regression function was estimated. For the selected groups the receiver operating characteristic (ROC) curves were obtained and the area under curve (AUC) was calculated with 95% confidence intervals according to the nonparametric method of DeLong. A *P* value of <0.05 was considered as statistically significant.

## 3. Results

When analyzing all patients regardless of their hormonal status, mean serum HE4 levels in BRCA1 carriers were significantly lower than in the remaining groups (uterine myomas *P* = 0.0138; noncancer ovarian cysts *P* = 0.0001; adnexitis *P* = 0.0079; endometrial cancer *P* = 0.0000; other gynecological cancers *P* = 0.0000; endometrial polyps *P* = 0.0023; metastatic tumors *P* = 0.000579; ovarian cancers *P* = 0.0000), with the exception of benign epithelial ovarian tumors (*P* = 0.1834). In postmenopausal women, statistically significant differences were observed only in comparisons with groups of patients with oncological diagnoses (endometrial cancers *P* = 0.0000; ovarian cancers *P* = 0.0000; metastatic tumors *P* = 0.0049; other gynecological cancers *P* = 0.0034). No differences were observed in the remaining groups of patients with benign gynecological disorders. In premenopausal BRCA1 mutation carriers, significantly lower serum HE4 levels were observed in comparison to patients with ovarian cancer (*P* = 0.0000), other gynecological cancers (*P* = 0.0002), uterine myomas (*P* = 0.0021), noncancer ovarian cysts (*P* = 0.0000), and adnexitis (*P* = 0.0035). No differences in mean HE4 levels were observed between BRCA1 mutation carriers and patients with endometrial polyps (*P* = 0.0669) or benign epithelial tumors (*P* = 0.6287). No comparisons were made to the group of endometrial cancer patients due to the low number of diagnosed cases in the premenopausal population.

Mean CA125 levels in BRCA1 mutation carriers were statistically different as compared to all study groups with the exception of endometrial polyps. Similarly, to HE4 levels, CA125 levels were the lowest in genetically burdened patients among all the study groups.

Table 2 presents mean serum HE4 and CA125 levels in ovarian cancer patients and other study groups. We demonstrated that serum HE4 and CA125 levels are in most cases statistically higher in ovarian cancer patients as compared to the remaining study groups. Lack of differences was demonstrated only in premenopausal patients with pelvic inflammatory disease (both HE4 and CA125), postmenopausal women diagnosed with endometrial cancer (HE4), and patients with ovarian metastatic tumors (HE4 and CA125 only).

Mean HE4 and CA125 levels were also compared between individual subgroups.  Table 3 lists the means, median, and ranges of the measured concentrations. We observed statistically higher serum HE4 levels in vulvar cancer patients (90.62 pmol/L) as compared to endometrial cancer patients (56.13 pmol/L); *P* = 0.0106 as well as statistically higher serum HE4 levels in patients with serous (720.67 pmol/L) and endometrial (419.07 pmol/L) ovarian cancers as compared to mucinous type of ovarian cancers (136.97 pmol/L) with significance levels of *P* = 0.0070 and *P* = 0.0431, respectively. No differences in HE4 or CA125 levels were observed in the remaining cases.

**Figure 1:**
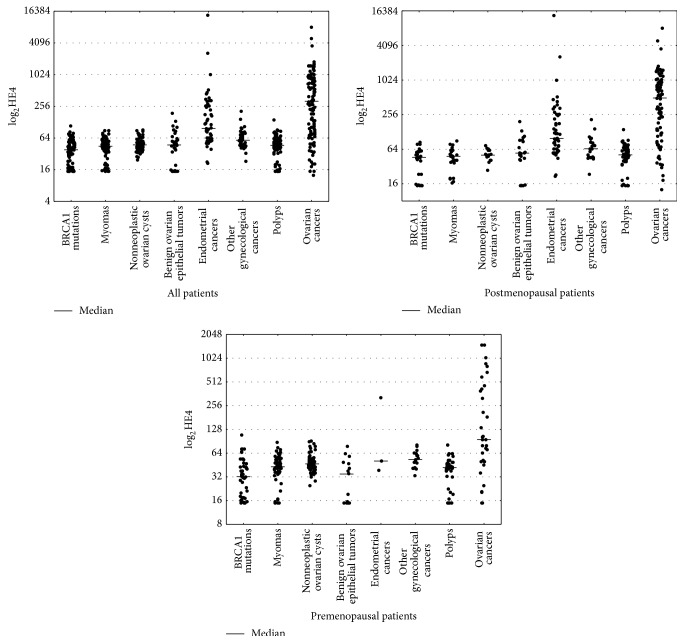
Scatterplot of serum HE4 levels by histopathological classifications.

Figure 1 presents distribution of HE4 results within the study groups. It is evident that median HE4 concentrations were the highest for ovarian cancers (409.75) followed by endometrial cancers (97.3), other gynecological cancers (58.15), noncancer ovarian cysts (47.45), benign epithelial tumors (47.27), endometriosis (46.5), myomas (44.65), and BRCA1 mutation carriers (38.25). The graph does not include patients with adnexitis (median 54.3) and metastatic tumors (median 209.9) due to the low number of cases. The pattern of serum HE4 levels in pre- and postmenopausal patients is similar, with the highest medians in the ovarian cancer and endometrial cancer groups and the lowest medians in the BRCA1 mutation groups.

**Figure 2:**
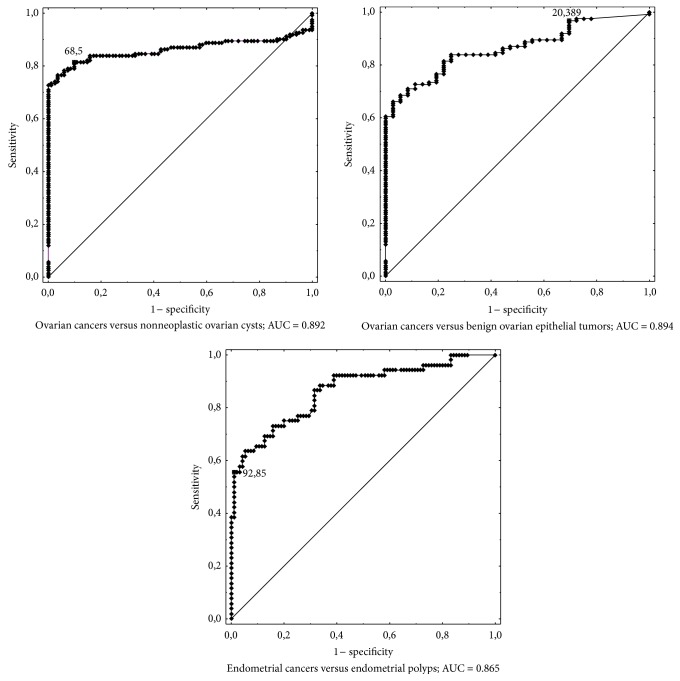
Receiver operating curves for HE4 in ovarian and endometrial cancer.

ROC curves presented in Figure 2 illustrate the usefulness of HE4 as a diagnostic assay. The relative area under the curve was 0.892 for differentiation of ovarian cancer from benign nonneoplastic ovarian cysts, 0.894 for differentiation of ovarian cancer from benign epithelial tumors, and 0.865 for differentiation of endometrial cancer from endometrial polyps. The respective ROC AUCs for CA125 were 0.932, 0.936, and 0.782. The differences between the ROC AUCs for CA125 and HE4 were statistically significant at *P* = 0.0148 for ovarian cancers versus noncancer ovarian cysts, *P* = 0.0076 for ovarian cancers versus benign epithelial tumors, and *P* = 0.0062 for endometrial cancers versus endometrial polyps.

Statistically significant correlations between the serum levels of CA125 and HE4 were observed in the ovarian cancer group (*r* = 0.3547,  *P* = 0.0001), endometrial cancer group (*r* = 0.7986,  *P* = 0.0011), and benign epithelial tumor group (*r* = 0.3629, *P* = 0.0349). In the remaining groups, no correlations were found between CA125 and HE4 levels.

A detailed analysis of BRCA1 carriers showed ovarian and fallopian tube cancer diagnosed in the postoperative material in 3 out of 83 cases (3.6%). Patient 1, aged 50, postmenopausal, had reported prophylactic surgery while being asymptomatic. Preoperative markers are CA125: 63.9 U/mL and HE4: 44.0 pmol/L. Laparoscopic hysterectomy and adnexectomy were performed as a part of prophylactic surgery. Intraoperatively, both ovaries were bilaterally unremarkable; no ascites or peritoneal spread was observed. Postoperative examination revealed a poorly differentiated (G3) serous cancer involving the entire left ovary. Right ovarian tissue is unremarkable. Another surgery was carried out including the full ovarian cancer procedure. Postoperative material revealed metastases only within para-aortic lymph nodes; patient was classified as FIGO stage III. Patient 2, age 41, clinically asymptomatic, had reported prophylactic surgery. Preoperative markers are CA125: 14.0 U/mL and HE4: 79 pmol/L. Bilateral laparoscopic adnexectomy was carried out with no macroscopic lesions identified within the adnexa. A G2 serous cancer was diagnosed in postoperative material (20% of ovary involved). The contralateral ovary and bilateral fallopian tubes were free of tumor infiltration. Another surgery including hysterectomy, adnexectomy, and para-aortic and iliac lymphadenectomy was performed. No cancer cells were identified in any postoperative specimen, confirming the very early clinical stage of the disease (FIGO I). Patient 3, age 39, was also asymptomatic. Preoperative markers are CA125: 39.4 U/mL and HE4: 32.4 pmol/L. Laparoscopic hysterectomy and adnexectomy were performed; ovaries and fallopian tubes were macroscopically unremarkable. Postoperative histopathological examination revealed G1 cancer of a fallopian tube with massive exfoliation of cancer cells to the tubal lumen with focal infiltration of the perivascular parenchyma; features of intraepithelial neoplasm within the other tube. Ovaries were bilaterally unremarkable. Subsequent surgery consisted of hysterectomy and omentectomy as well as para-aortic and iliac lymphadenectomy. No cancer cells were identified in the postoperative material. The final diagnosis was G1 fallopian tube cancer, FIGO stage I.

Twenty-four (30%) of 80 patients with BRCA1 mutations had been previously diagnosed and treated for breast cancer. No patient had an active disease upon being qualified for the study. All patients were in remission. Mean HE4 level in this subgroup was 35.9 pmol/L. Over several years of follow-up, we observed 4 additional breast cancer cases occurring within the period of 6 months to 3 years after the prophylactic surgery. One patient suffered a breast cancer relapse 6 months after surgery. One patient developed primary peritoneal cancer 8 months after the prophylactic surgery, accounting for 1.25% of the entire study group. At the time of prophylactic surgery, the HE4 level was 15 pmol/L. At the time of diagnosis of primary peritoneal cancer, it was 454.3 pmol/L.

## 4. Discussion

The studies aimed at the development of an ovarian cancer screening test have been ongoing for several decades. After thousands of women were studied none of the methods proposed to date could meet the criteria of a screening test. Methods attempted to date were inefficient in reducing the mortality due to ovarian cancer or in increasing the percentage of women in whom the disease is diagnosed at an early stage. In addition, a high percentage of false positive results leads to unnecessary surgeries which are associated with complications and stress [18]. Only the group of patients genetically predisposed to the ovarian cancer benefits from prophylactic screening which should lead to prophylactic salpingooophorectomy at an appropriate age [16, 17]. The assay that has been used most commonly in the screening of patients genetically predisposed to ovarian cancer is determination of serum CA125 levels and annual transvaginal ultrasound scans. Recent studies conducted in a group of patients at high risk of ovarian cancer showed that the risk of ovarian cancer algorithm (ROCA), consisting of serial determinations of CA125 levels, individualization of these levels for each patient, and stratification of patients into ovarian cancer risk strata determining further management may be of high clinical usefulness [19].

The researches expect new possibilities for the ovarian cancer screening to be offered by the novel cancer marker, HE4, used to date along with CA125 mainly in the diagnostics of pathological developments within the adnexa [1–10]. To date, only a few articles assessing the levels of this marker in a group of females at high risk of ovarian cancer were studied [20–22]. Shah et al. [20] demonstrated that a mean HE4 level was not significantly different in healthy females at high and medium risk of ovarian cancer, although the mean values were somewhat lower in the high-risk group. The CA125 levels were statistically lower in high-risk subjects. According to the authors, the high-risk group consisted of patients with a positive family history (at least two ovarian or breast cancers in first- or second-degree relatives), BRCA1 carriers, and Ashkenazi Jews with a positive family history (one ovarian or breast cancer in a first-degree relative or two cancers in second-degree relatives). When assessing the diagnostic usefulness of CA125 and HE4 levels in ovarian cancer, the researchers determined that the area under the ROC curve was not statistically different when the control group consisted of patients with medium (CA125: 0.939, HE4: 0.928) or high (CA125: 0.939, HE4: 0.931) risk of ovarian cancer. Anderson et al. [21] evaluated the potential for predicting ovarian cancer using a symptom index, CA125, and HE4 as a multimodality, multistage screening program. When analyzing different combinations of these three parameters, the researchers came to a conclusion that the presence of 2 of 3 analyzed parameters as the first line of ovarian cancer is characterized by specificity of ca. 98.5%. They also suggested that inclusion of transvaginal ultrasound scan as the second line of screening might increase the specificity and PPV to the acceptable level. Patients at high risk of ovarian cancer were also the subjects of the study conducted by Urban et al. [22]. The authors assumed that HE4 might be useful in ovarian cancer screening, as the reference standards presented to date are contradictory. In their publication they decided to determine the reference standards of HE4 for patients with BRCA 1 mutations in age above 25 and for patients with family history of breast or ovarian cancer in age above 35. Serial analyses were conducted on individual HE4 and CA125 levels depending on the age, race, hormone replacement therapy status, contraception status, smoking status, and history of oophorectomy or tubal ligation. The study results showed that HE4 was lower in black females and higher in smokers and markedly increased with age, particularly after the age of 55, which should be taken into account when deciding upon screening tests based on HE4 determinations. In our studies, mean HE4 levels were assessed in a group of patients at the highest risk of ovarian cancer, that is, in patients diagnosed with BRCA1 gene mutation. According to current estimates, 5–15% of all ovarian cancers are associated with germinal mutations, with 90–95% of these consisting of mutations within BRCA1/2 genes [23]. The risk of ovarian cancer in the overall population is about 1.6% while it reaches up to 60% in BRCA1 carriers [23]. As shown by our analysis of 80 BRCA1 carriers, mean levels of the marker in the study populations were significantly lower than in the group of females with benign gynecological disorders (functional and nonneoplastic cysts, uterine myomas, and endometrial polyps) in the whole examined groups and premenopausal patients. There were no significant differences in the mean age of patients in the study groups, which is particularly important as HE4 levels are age-dependent. In our study we do not analyzed group of healthy women. However, comparing our results with those of healthy women cited by Roche in the summary of product characteristics (http://www.cobas.com/content/dam/cobas_com/pdf/product/Elecsys HE4 Human Epididymal Protein 4/HE4 fact sheet.pdf) also shows a trend towards lower values of serum HE4 levels in carriers of BRCA 1 compared with healthy women. To make this analysis we divided our patients with the same age range as quoted by Roche study and compared median values. The results were as follows: in the under 40 years of age median value of HE4 in carriers was 41 pmol/L and 42 pmol/L, in healthy women, between 40–49 years of age, respectively, it was 31.1 pmol/L and 44.3 pmol/L, in the range of 50–59 years, it was 44.7 pmol/L and 47.9 pmol/L, and in the range 60–69 years in carriers of BRCA 1 and healthy women values were identical (55 pmol/L). Of course, due to the lack of source data for healthy women statistical comparisons were not performed which is a defect of this analysis. None of the three papers cited above [21, 22] assessed HE4 levels in a group of BRCA1 gene mutation carriers only. BRCA mutation carriers were included in the high-risk groups along with females with family history but no genetic mutations. Therefore, this report is the first to present the values of HE4 levels in a group of female patients with BRCA1 gene mutation. The values in high-risk patients as reported by Urban et al. [22] were higher than those observed in our study. In the group of premenopausal women with the mean age of 43 years, the mean HE4 level measured in our study was 35.7 pmol/L as compared to 27.7 pmol/L reported by the aforementioned authors; the respective values in postmenopausal women were 48.3 pmol/L in our study and 31.3 pmol/L in the study by Urban et al. [22]. However, one should take note of different laboratory methods being used in Roche versus Abbott studies. In the study by Urban et al. [22], patients with BRCA1/2 mutations accounted for 17.7% (138 cases) of the population included in the analysis. It appears that when deciding upon the future use of HE4 determination in ovarian cancer screening, the low values measured in patients with germinal BRCA1 mutation should be taken into account, which obviously requires further research in a significantly larger population of females.

Occult ovarian cancer was diagnosed by histopathological examination in 3 out of 83 (3.6%) women undergoing surgery. In either of these three cases, the ovaries were macroscopically unremarkable. All 83 surgeries were carried out by means of laparoscopic technique. The surgeries included bilateral adnexectomies in most cases and subtotal hysterectomies in selected cases when requested by the patient or when they are due to uterine myomas. The incidence of occult ovarian/fallopian tube cancers in patients having undergone prophylactic surgeries for BRCA1 gene mutation is varied [24–26] and ranges from 1.9% [25] to 16.2% [26]. Similarly at our center, most surgeries are carried out by laparoscopic technique which appears to be safe and worth recommendation [27, 28]. The wide distribution of results regarding the diagnosis of latent ovarian cancer in BRCA1 gene mutation carrier is most probably due to different sizes of the study samples and average ages of patients when undergoing the prophylactic surgery (the populations of patients in cited studies ranged from 37 [26] to 374 [29] females). Regardless of the cited incidence of occult ovarian cancer, particular care is recommended during prophylactic surgeries consisting of delicate removal of ovaries from the abdominal cavity, preferably using endobags, so as to avoid potential cancer spread as well as in appropriate preparation of the team of pathologists who should be aware of the very small size of possible neoplastic foci within the ovaries of patients at high risk of cancer. Of particular note is the fact that each of the 3 patients with latent ovarian cancer in our study presented with discrete elevation of one of the two markers. Therefore, one might consider a management approach involving each patient qualified for prophylactic surgery being subjected to HE4 and CA125 determination; in case of elevation of one of the markers, the range of diagnostic procedures should be widened, intraoperative examination included.

HE4 is a relatively novel tumor marker used in the diagnostics of pathological lesions within the adnexa. Its sensitivity is very similar to that of CA125 while its specificity is much higher [2, 8, 10, 14, 30]. Despite reports that differ in the assessment of diagnostic usefulness of HE4, suggesting either CA125 [8, 30] or HE4 [2, 10, 14] as the more useful marker, HE4 has already become an established marker in the diagnostics of ovarian cancer. Our results confirm the high diagnostic value of HE4 despite the fact that better results were obtained for CA125. Both markers based on AUC values for the ROC curves meet the criteria of a good diagnostic test and similar results have been published by other authors [2, 10], postulating simultaneous use of both markers in the diagnostics of ovarian cancer, for example, with employing the ROMA algorithm.

No cancer marker of potential importance in preoperative diagnostics has been found to date for endometrial cancer. Based on the research conducted in recent years, it appears that this vacancy may be filled by HE4 [31–37]. In 2013, Jiang et al. [37] demonstrated very high tissue expression of HE4 in endometrial cancer tissues. This was confirmed by Li et al. [31] who additionally demonstrated that high values of the marker's levels are correlated with tumor size, clinical stage, and depth of myometrial involvement while tissue overexpression has an influence on the progression of cancer. The authors reported the clinical usefulness of HE4 in both early [38] and advanced stages of endometrial cancer [33]. The results of our studies are consistent with those obtained by other authors and confirm that endometrial cancer is accompanied by elevated HE4 levels. The diagnostic value of HE4 is confirmed by high AUC value (0.865) calculated for endometrial cancer relative to endometrial polyps. The value was statistically significantly higher as compared to the ROC-AUC value for CA125 (0.651). In the studies by Moore et al. [38], ROC-AUC in endometrial cancer was 0.671 for CA125 and 0.787 for HE4 and *P* = 0.0007. The authors assessed endometrial cancers of all stages as compared to healthy volunteers. Similar results were presented by Saarelainen et al. [33], with ROC-AUC amounting to 0.76 for HE4 and 0.65 for CA125. How can we therefore make use of the measurements of serum HE4 levels in endometrial cancer? While preoperative diagnostics putting forth the suspicion of endometrial cancer is relatively straightforward due to typical symptoms, risk factors, and transvaginal ultrasound results, a method helpful in making decisions regarding the surgical treatment is still being sought for. Iliac and para-aortic lymphadenectomy is recommended in patients with high-risk factors (G3, tumor size of more than 2 cm, involvement of vascular spaces, and deep involvement of myometrium). Unfortunately, data on these risk factors are usually unavailable at the moment of decision regarding the surgical treatment. The statistically significant high concentrations of HE4 in endometrial cancer and as observed in our study as well as the correlation of these concentrations with high-risk factors reported by other authors [31, 33, 38] should warrant the conduct of further prospective studies.

## 5. Conclusion

Patients with BRCA1 gene mutations are characterized by relatively low serum HE4 levels. The trend is particularly evident in younger, premenopausal females. Determination of CA125 and HE4 in every patient carrying the BRCA1 gene mutation and undergoing prophylactic surgery appears justifiable. Even the slightest elevation in HE4 or CA 125 levels in these patients should significantly increase oncological alertness. The HE4 marker is a valuable tool not only in differentiation of malignant and benign lesions within the adnexa, but also in differentiation of malignant and benign disorders of the uterine endometrium.

## Figures and Tables

**Table 1:** Patient (groups) demographics.

	*n*	Age mean	Age range
All groups	619	51.09	18–92

Group A: BRCA 1 mutation			

All	83	47.9	34–64
Premenopausal	53	43.35	34–51
Postmenopausal	30	56.93	48–64

Group B: myomas			

All	90	47.10	25–79
Premenopausal	63	42.75	25–52
Postmenopausal	27	57.26	44–79

Group C: nonneoplastic ovarian cysts			

All	82	35.4	18–75
Premenopausal	66	30.39	18–53
Postmenopausal	16	56.06	54–75

Group D: inflammatory diseases			

All	9	34.67	18–43
Premenopausal	9	34.67	18–43
Postmenopausal	—	—	—

Group E: benign epithelial ovarian tumors			

All	35	48.77	18–88
Premenopausal	15	29.27	18–48
Postmenopausal	20	63.4	54–88

Group F: endometrial cancers			

All	55	66.7	54–92
Premenopausal	2	43.5	43–44
Postmenopausal	53	68.3	54–92

Group G: other gynecological cancers			

All	38	53.84	19–92
Premenopausal	17	37.41	19–51
Postmenopausal	21	67.14	52–92

Group H: endometrial polyps			

All	95	52.92	19–83
Premenopausal	40	39.5	19–50
Postmenopausal	55	62.67	49–83

Group M: other cancers (metastatic ovarian tumors)			

All	8	62.38	51–78
Premenopausal	—	—	—
Postmenopausal	8	62.38	51–78

Group O: ovarian cancers			

All	124	58.23	30–90
Premenopausal	33	44.64	30–65
Postmenopausal	91	63.2	48–90

**Table 2:** Average concentrations and comparisons of serum HE4 and CA125 levels between patients with ovarian cancer and the other study groups.

Group	HE4 [pmol/L] mean/range median/95% confidence intervals 95th percentile	CA 125 [mIU/mL] mean/range median/95% confidence intervals 95th percentile	HE4 [pmol/L] mean/range median/95% confidence intervals 95th percentile	CA 125 [mIU/mL] mean/range median/95% confidence intervals 95th percentile	HE4 [pmol/L] mean/range median/95% confidence intervals 95th percentile	CA 125 [mIU/mL] mean/range median/95% confidence intervals 95th percentile
	All patients		Premenopausal patients		Postmenopausal patients	

Ovarian cancers	633.76/15–8160	1480/9.12–10000	308.71/15.57–1500	615.58/25.32–4638.8	751.6/15–8160	1049.7/9.12–10000
	319.36/460.9–806.6	409/671.1–1197.3	95.40/162.2–455.2	354/292.5–938.7	499.2/525.5–977.7	432/710.8–1388.6
	1516.5	4203	1500	2096	1655	4459.7

versus BRCA1 mutations	38.78/15–152.7	21.09/2.98–223	35.73/34–51	23.2/2.98–223	48.3/15–152.7	17.21/3–36.3
	38.25/34.1–43.5	15.64/13.2–28.9	44/30–41.4	15.78/11.5–34.8	47.3/37.1–59.6	15.25/13.2–21.3
	77	30.7	72.3	28.2	78.2	30.7

*P*	0.0000	0.0000	0.0000	0.0000	0.0000	0.0000

versus myomas	45.07/15–88.8	27.49/9.3–173.6	44.12/15–87.8	27.49/9.3–173.6	47.3/16.34–88.8	20.18/10.3–60.2
	44.65/41.6–48.5	18.8/16.9–34.4	43/40.2–48	43/16.2–38.8	48.1/39.9–54.7	16/9.7–30.7
	74.9	58.5	67.7	56.5	77.8	60.2

*P*	0.0000	0.0000	0.0000	0.0000	0.0000	0.0000

versus nonneoplastic ovarian cysts	49.85/24.8–90.4	36.75/4.1–377	49.45/24.8–90.4	41.62/4.1–377	51.47/27.50–73.9	16.67/4.54–82.1
	47.45/46.9–52.8	17.15/24.7–48.8	46.75/46.6–52.8	19.9/27–56.2	50.5/45.2–57.8	7.55/6.8–26.5
			78	124	73.9	82.1

*P*	0.0000	0.0000	0.0000	0.0001	0.0000	0.0000

versus diseases	85.5/39–269.8	201/2.4–715.9	85.5/39–269.8	201/2.4–715.9	—	—
	54.3/26.1–144.9	85/5.9–396.1	54.3/11.7–142.5	85/3.7–442		
	269.8	715.9				

*P*	0.0027	0.0027	0.0902	0.1302	—	—

versus benign epithelial ovarian	52.54/15–191.1	31.08/3.86–186.72	34.58/15–78.2	22.51/8.56–56.40	65.36/15–191.1	36.8/3.86–186.7
	47.27/39.5–65.6	22.2/19.9–42.2	34.8/22.9–46.2	21.4/13.4–31.6	54.55/45.6–85.1	24.8/18.9–54.6
tumors	131.5	64.7	78.2	56.4	131.5	64.7

*P*	0.0000	0.0000	0.0001	0.0000	0.0000	0.0000

versus endometrial cancers	477.53/21.37–13762	134.62/11.6–1200	184.45/39.1–325.8	25.75/28.4–22.2	498.3/21.37–13762	142.7/11.6–1200
	97.3/−56.1–1011.1	26.6/−37.8–306.9	221.55/−264.5–541.6	25.28/7.8–14.6	98.3/−68.5–1065	29/−42.9–328
	1017	1200.3	325.8	22.2	1017	1200.3

*P*	0.0007	0.0001	—	—	0.0643	0.0001

versus other gynecological cancers	65.98/23.16–208.5	37.34/8.2–101.5	54.69/32.9–81.1	41.29/8.2–98.5	75.12/23.16–208.5	33.82/11.1–101.5
	45.9/55.1–76.9	13/19.9–54.8	53.3/47.1–61.6	17.1/7.9–74.6	65.1/56.3–93.9	17.6/10.9–56.7
	145.7	101.5	81.1	98.5	145.7	101.5

*P*	0.0000	0.0000	0.0085	0.0006	0.0000	0.0000

versus endometrial polyps	48.08/15–141.8	21.66/2.6–182.8	41.31/15–81.30	17.16/16.99–29	53/15–141.8	23.35/2.6–182.8
	46.5/43.7–52.5	13.7/5.4–37.9	42/35.9–46.7	18.2/9.3–25.1	51/46.7–59.4	12.5/0.35–46.4
	83.6	34.2	72.1	29	88.9	182.8

*P*	0.0000	0.0000	0.0000	0.0002	0.0000	0.0000

versus other cancers	236.51/12.1–580.1	615.4/21.8–3091	—	—	236.51/15–580.1	615.4/21.8–3091
	209.9/88.2–384.2	257.3/−238.5–1469.3			209.9/49.7–389	257.3/−332.8–1680.8
	580.1	3091			580.1	3091

*P*	0.2790	0.2858	—	—	0.0798	0.2471

**Table 3:** Average concentrations of serum HE4 and CA125 levels in the different subgroups.

Subgroup	HE4 [pmol/L] mean/range median/95% confidence intervals 95th percentile	CA 125 [mIU/mL] mean/range median/95% confidence intervals 95th percentile	HE4 [pmol/L] mean/range median/95% confidence intervals 95th percentile	CA 125 [mIU/mL] mean/range median/95% confidence intervals 95th percentile	HE4 [pmol/L] mean/range median/95% confidence intervals 95th percentile	CA 125 [mIU/mL] mean/range median/95% confidence intervals 95th percentile
	All patients		Premenopausal		Postmenopausal	

Nonneoplastic ovarian cysts:						

Endometriosis	49.74/27.5–90.4	62.12/8.8–377	49.14/33.8–90.4	65.71/8.8–377	53.93/27.5–73.90	36.93/8.8–82.1
	46.05/43.7–55.7	41.7/30.5–93.8	45.4/43.2–55.1	43.9/29.9–101.5	60.4/−5.3–113.2	19.9/−61.2–135.1
	73.9	131.2	66.9	131.2	73.9	82.1

Teratomas	47.12/31.9–71.4	21.32/17.3–54.35	46.89/31.9–71.4	22.79/7.8–54.35	48.4/47.1–50.5	12.93/7.3–16.4
	47.45/43–51.2	16.45/15.3–27.3	47.3/41.9–51.8	18.5/15.9–29.6	47.6/43.8–52.9	15.1/0.7–25.2
	63.5	53.1	71.4	54.4	50.5	16.4

hemorrhagic corpus luteum cysts	47.33/28.3–84.7	36.5/4.1–111.4	46.74/24.8–88.6	41.16/6.7–274.8	49.9/37.8–61.4	16.3/7.5–29
	44.3/39.8–54.9	15.35/29.9–58.7	44.2/37.7–55.8	16.1/−3.7–86.2	50.5/20.6–79.2	12.4/−11.7–44.3
	88.6	274.8	88.6	274.8	61.4	29

Paraovarian cysts	54.28/24.8–88.6	23.31/6.7–274.8	55.15/28.3–84.7	29.64/4.10–111.40	52.4/40.5–66.1	9.73/4.54–16.7
	51/47.6–60.9	11.95/10.4–36.2	48.2/45.7–64.6	13.5/11.1–48.2	51.7/42.9–61.9	7.9/5.6–13.9
	78	88	84.7	111.4	66.1	17.7

Benign epithelial ovarian tumours:						

Serous cystadenomas	43.79/15–107	42.48/8.56–186.72	25.63/15–78.2	22.54/8.56–55.4	61.94/15–107	62.42/12.1–186.72
	28.51/22.3–65.3	26.88/11.2–73.8	15/−1.39–52.66	16.68/3.3–41.8	61.17/27.7–96.2	43.9/−4.3–129.2
	107	186.7	78.2	55.4	107	186.7

Mucinous cystadenomas	67.21/15–191.1	29.12/3.86–64.67	40.99/19.2–58.4	26.23/11.3–56.40	84.69/15–191.1	31.04/3.86–64.67
	49/41.2–93.3	22.2/18.6–39.6	42.27/26.7–55.3	22/9.9–42.5	90.82/43.4–126	23.12/14.44–47.64
	191.1	64.7	58.4	56.4	191.1	64.7

Cystadenofibroma	39.75/15–62.6	17.67/6.35–40	39.67/15–62.6	11.23/8.86–13.6	39.8/15–55.8	19.81/6.35–40
	44.62/24.8–54.7	13.55/8.5–26.9	41.41/−19.6–98.9	11/−18.9–41.3	49/19.4–60.2	16.29/7.19–32.44
	62.6	40	62.6	13.6	45.9	49.2

Other gynecological cancers:						

Gonadal tumors	42.9/23.5–59.8	37.1/29.4–49.2	59.78/59.78	32.8/32.8	34.53/23.16–45.9	39.3/29.4–49.2
	45.9/−2.9–88.9	32.8/5.4–48.9	59.78/—	32.8/—	34.53/−109.9–179	39.3/
	59.8	49.2	—	—	45.8	49.2

Vulvar cancer	90.62/32.9–208.5	13.32/8.2–17.6	46/32.9–59.1	10.9/8.2–13.6	99.54/45.65–208.5	15.03/11.1–17.6
	83.79/60–121.2	13.75/6.3–20.4	46/−120.5–212.5	10.9/−1.2–15.8	90.65/65.5–133.6	16.4/6.44–23.5
	208.5	17.6	59.1	8.2	208.5	17.6

Cervical cancer	56.13/40.4–81.1	44.45/12.2–101.5	55.57/40.4–81.1	42.56/12.2–98.5	57/42.7–74.1	42.02/13–101.5
	53.3/50.6–61.7	22.7/20.6–68.1	52.6/47.7–63.4	17.1/−2.8–88.1	57.2/47.6–66.4	26.8/−3.78–87.8
	78.1	101.5	81.1	98.5	74.1	101.5

Ovarian cancers						

Serous ovarian cancer	720.67/15–8160	1049.31/9.12–10000	352.65/15–1500	695.29/25.32–4638.8	865.8/18.3–8160	1188.92/9.12–10000
	414.3/509.4–931.9	445/730.4–1368.2	119.75/184–521.3	382.32/320.4–1070.2	639/583.6–1148.1	500/768.9–1608.9
	1655	4638.8	1500	2096	1796.6	5109.8

Mucinous ovarian cancer	136.97/15–538.66	140.56/11.3–600	48.65/45.4–51.9	26.7/26.4–27	166.4/12.58	178.52/11.3–600
	57.35/−11.2–285.2	58.81/−25.2–306.4	48.65/7.35–89.9	26.7/22.9–30.5	76.35/−43–375.8	87.16/−51.8–408.8
	538.7	600	51.9	27	538.7	600

Endometrioid ovarian cancer	419.07/46.11–1235	788.85/41.5–2996.8	64.5/64.5	500/500	454.53/46.107–1235	817.74/41.5–2996.8
	273/151.7–686.5	397/136.4–1441.4	64.5/—	500/—	289.3/167.8–741.3	369.75/88.9–1546.6
	1235	2996.8	—	—	1235	29996.8

Clear cell ovarian cancer	255.77/49.49–849.9	359.1/95.8–991.9	75.78/49.49–102.07	146.3/95.8–196.8	345.76/76.733–849.9	465.5/96.4–991.9
	119.19/−65.9–577.4	193.75/−17.4–735.6	75.78/−258.3–409.8	146.3/-495.4-787.9	228.21/−213.8–905.4	386.85/−185.8–1116.8
	849.9	991.9	102.1	196.8	849.9	9991.9

## Abbreviations

ROMA: Risk of Malignancy Algorithm
HE4: Human epididymal protein 4
CA 125: Cancer antigen 125
FSH: Follitropin
ROC: Receiver operating characteristic
AUC: Area under curve
ROCA: Risk of ovarian cancer antigen
PPV: Positive predictive value
NPV: Negative predictive value.
